# Supplementary material for: Ascertaining provider-level implicit bias in electronic health records with rules-based natural language processing: A pilot study in the case of prostate cancer
Source: PLoS One. 2024 Dec 30;19(12):e0314989. doi: 10.1371/journal.pone.0314989 (PMC11684669; doi:10.1371/journal.pone.0314989)
Supplement: S1 Appendix — (DOCX) [file pone.0314989.s001.docx]

**APPENDIX: ­Ascertaining Provider-Level Implicit Bias Using Electronic Health Records**

1. **REGEX ONTOLOGIES**
2. *Active Surveillance (AS)*

(?<!(not\s))active surveillance(?![\w\s\(\)\.\,\-\']{0,2000}(ralp|rarp|benefit from surgical therapy|i would recommend radiation|proceed with prostatectomy|likely benefit from radiation therapy))

\bon (active surveillance|as\b)

\bwith (active surveillance|as\b)

\bundergo (active surveillance|as\b)

(?<!(not\s))recommend (active surveillance|as\b)

\bRecommended (active surveillance|as\b)

\bInitiate (active surveillance|as\b)

Good candidate for (active surveillance|as\b)

Candidate for (active surveillance|as\b)

Proceed with (active surveillance|as\b)

\bcontinue (active surveillance|as\b)

\bcontinued (active surveillance|as\b)

\bcontinuing (active surveillance|as\b)

understands the benefits and risks involved with active surveillance and wishes to proceed

benefit from (active surveillance|as\b)

1. *Molecular Biomarkers*

biomarker

\bclassifier\b

\bdecipher\b

\boncotype\b

\bonco\b

\bGPS\b

\bprolaris\b

\brisk score\b

\bgenomic score\b

1. *Urinary Function Evaluation*

continen

incontinen

\b(pad|pads)\b

nocturia

diaper

dripping

leakage

urge

\bstress\b

\bSymptom Score\b

\bIPSS\b

Epic

1. *Erectile Function Evaluation*

erecti

SHIM

Epic

The quality indicator was considered met if one or more clinical notes in a given patient’s EHR documentation contained language addressing a(n) (1) decision to initiate or continue AS, (2) consideration or utilization of prognostic prostate cancer biomarkers, (3) evaluation of urinary function either via survey or free-text, and (4) evaluation of sexual (i.e. erectile) function either via survey or free-text.

1. **GLOSSARY OF REGEX ONTOLOGIES**

**Active Surveillance**: A management strategy in prostate cancer involving active monitoring the course of disease, with regular prostate-specific antigen (PSA) testing, imaging, and prostate biopsies, with the expectation to intervene with curative intent if the cancer progresses.^1^

**RALP**: Robot-Assisted Laparoscopic Prostatectomy; is equivalent to RARP.

**RARP**: Robot-Assisted Radical Prostatectomy; is equivalent to RALP.

The following molecular biomarkers are tumor-based molecular assays that aid in risk stratification for patients with low or favorable intermediate risk prostate cancer:^1^

- **Decipher Prostate Genomic Classifier**^2^
- **Oncotype Dx Genomic Prostate Score**^3^
- **Prolaris Prostate Cancer Prognostic Test**^4^

**IPSS**: The International Prostate Symptom Score (IPSS), also known as the American Urologic Association Symptom Index, is a validated questionnaire to diagnose and measure the severity of urinary symptoms in men.^5^

**SHIM:** The Sexual Health Inventory for Men (SHIM), also known as IIEF-5 (International Index of Erectile Function) is a validated questionnaire to diagnose and measure the severity of sexual dysfunction in men.^6^

**EPIC-CP:** The Expanded Prostate Cancer Index Composite for Clinical Practice (EPIC-CP) is a validated tool for prostate cancer patients to assess health related quality of life after prostate cancer treatment; it evaluates both urinary and sexual function.^7^

1. **ANNOTATION AND VALIDATION OF REGEX ONTOLOGIES**

Of the 1,094 patients in our study population, 20 patients (associated with 373 notes) were randomly selected to generate a validation data set of random, representative unstructured clinical documentation based on a previously defined methodology of assessing regex ontology performance in which 20 patients were randomly selected per clinical site.^8^ The validation set subsequently underwent gold standard manual review and NLP-assisted annotation using Clinical Regex by two trained annotators. On average, annotators screened 101 and 423 notes in one hour with manual review and NLP-assisted annotation, respectively. Our ontologies possessed 100% precision and sufficiently high recall for all four quality indicators at both the note- and patient-level (**Supplementary Table 1**).

**Supplementary Table 1. Precision and Recall of Quality Indicators, Note- and Patient-Level**

|  | Precision (notes) | Recall (notes) | Precision (patient) | Recall (patient) |
| --- | --- | --- | --- | --- |
| Active surveillance | 100% | 100% | 100% | 100% |
| Molecular Biomarkers | 100% | 93.8% | 100% | 100% |
| Urinary Function | 100% | 77.9% | 100% | 93.3% |
| Erectile Function | 100% | 90% | 100% | 93.8% |

1. **CLINICAL REGEX EXAMPLES**
2. True positive example

The following examples were extracted by our designed regex algorithm; human annotators subsequently determined that these patients were in fact undergoing active surveillance.


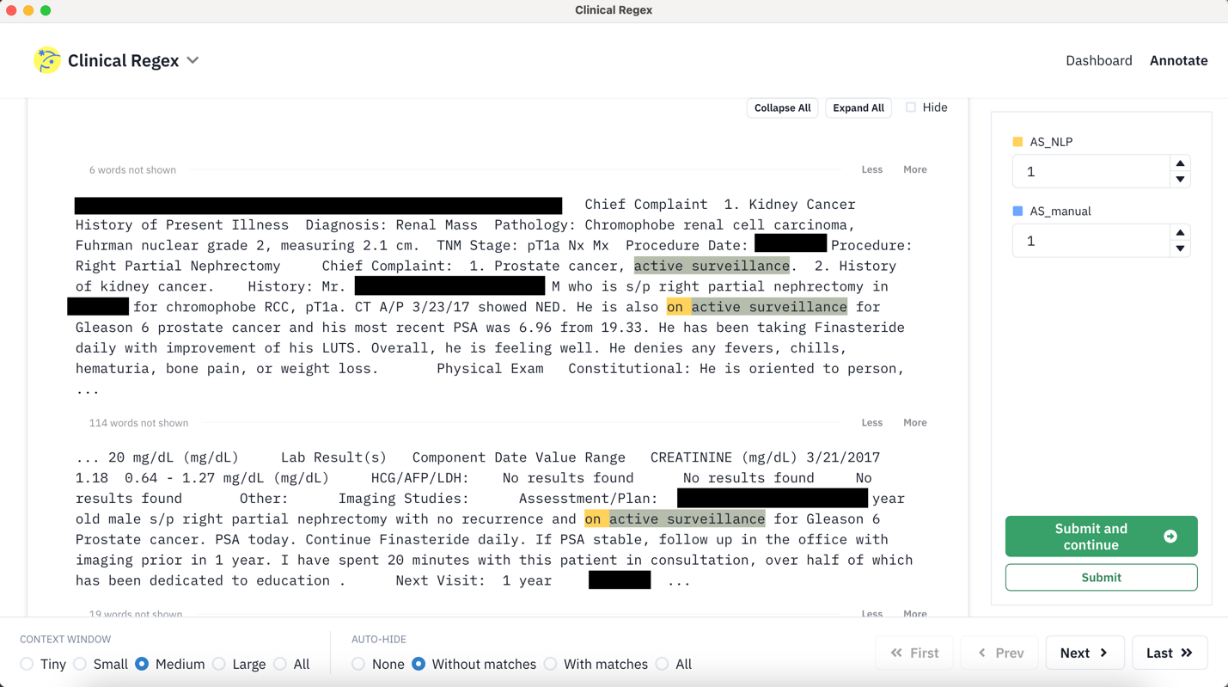


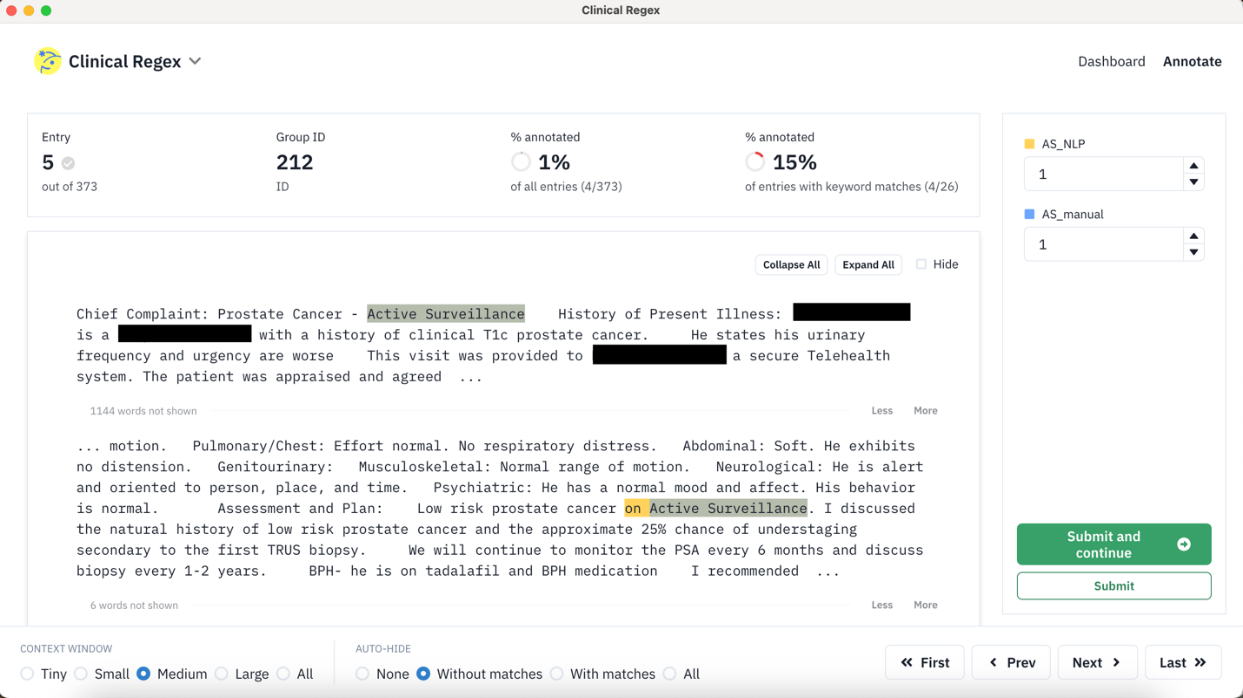


1. False positive example

In the following examples, the regex algorithm extracted these patients, however human annotators did not subsequently determine the patients as undergoing active surveillance. In the first example, all treatment options were discussed including active surveillance, however a determination was not made; in the second example, active surveillance referred to a kidney mass rather than prostate cancer.


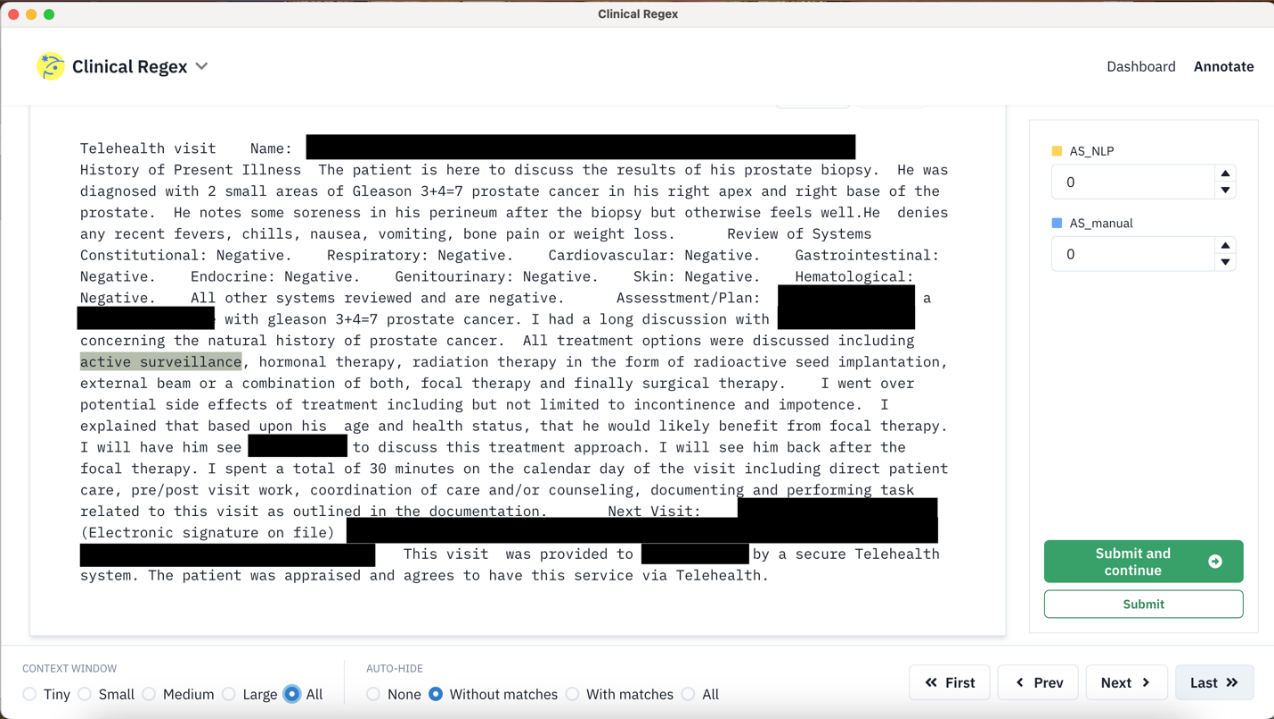


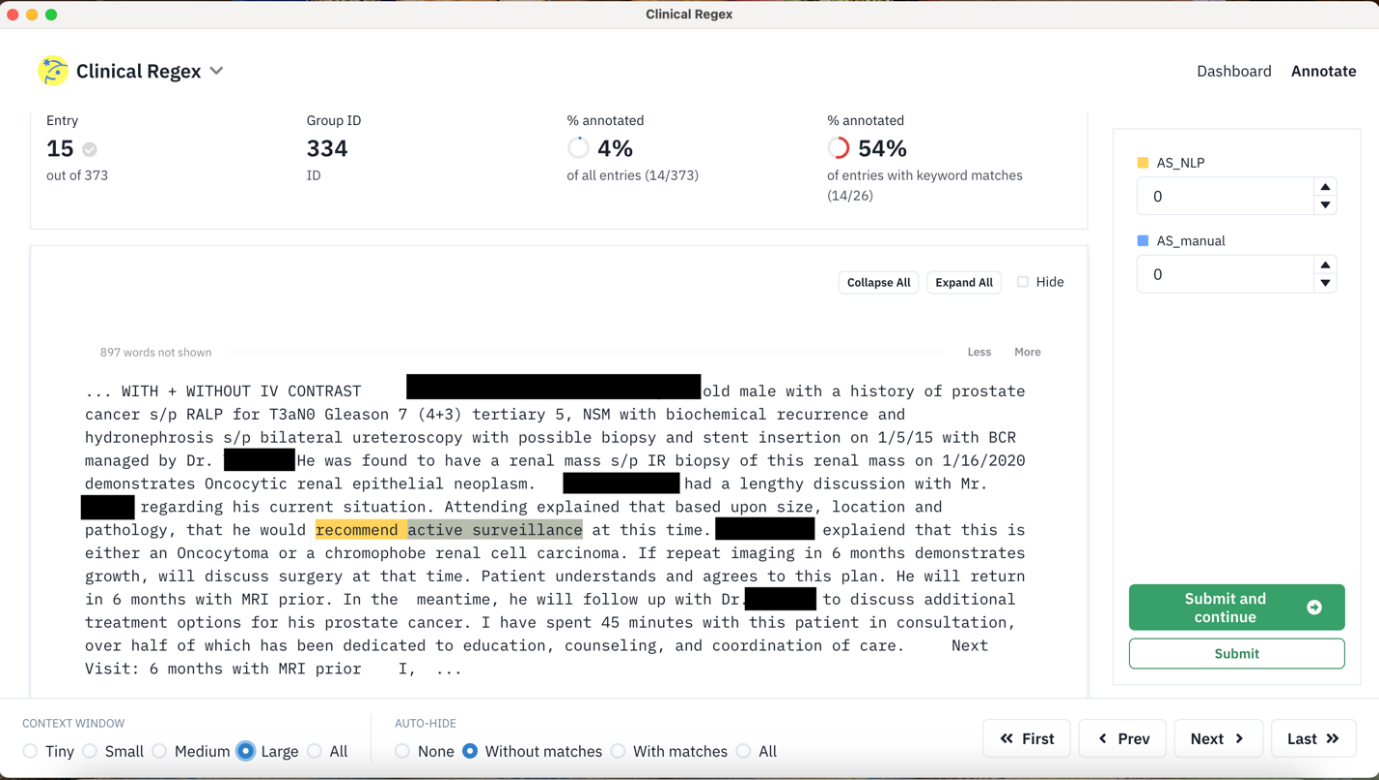


1. Regex algorithm – improving recall

We designed our regex algorithm to have flexible syntax to improve the recall of our model. In the following example, the regex algorithm extracted a patient on active surveillance using a common provider abbreviation “AS”.


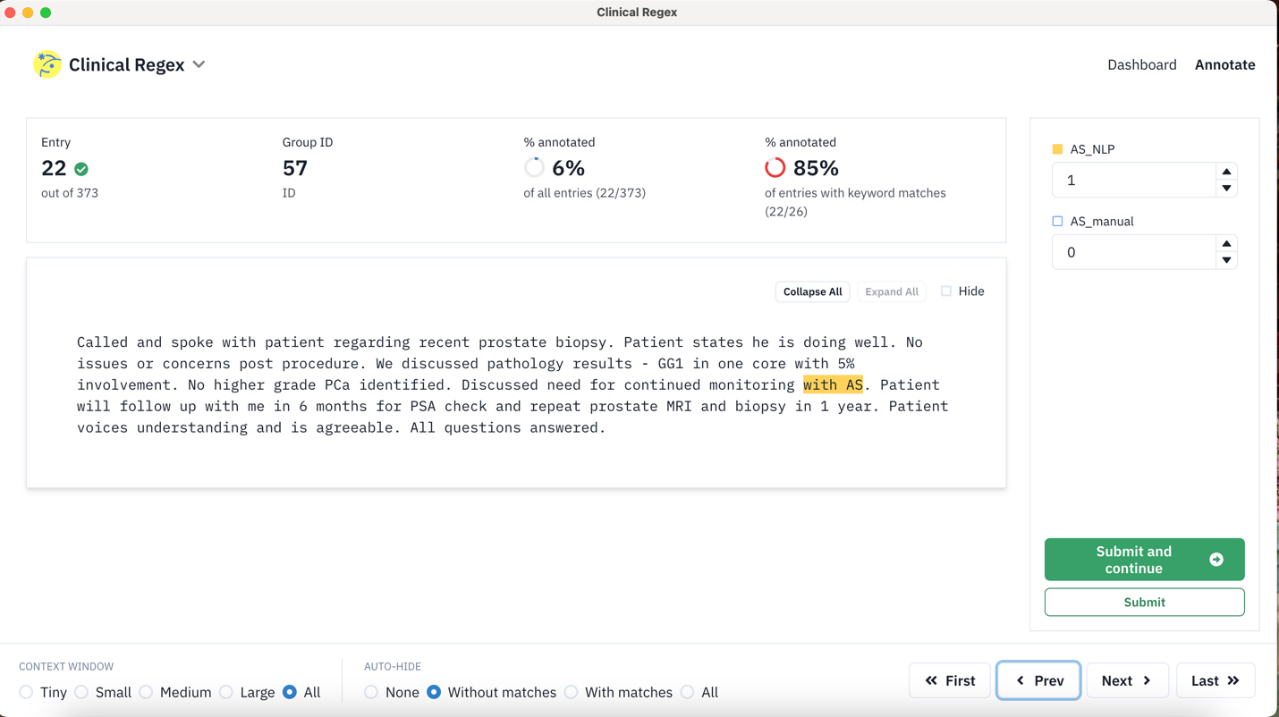


Additionally, we improved the flexibility of our model such that it would retrieve notes secondary to misspelling or from additional spaces or characters. In the following example, “active surveillance” contains 3 spaces between the words; in this specific case, the model did not retrieve this note as the patient underwent cryotherapy.


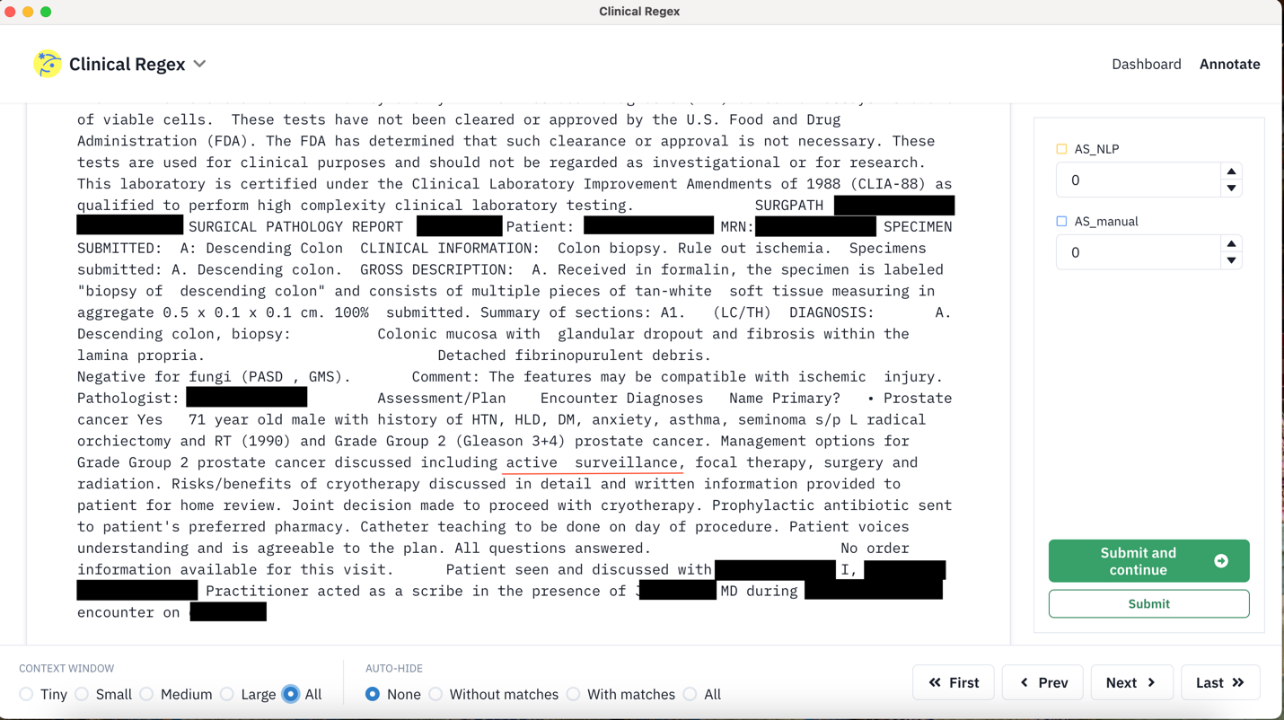


1. Regex algorithm – improving precision

We designed our regex algorithm to not extract mentions of active surveillance when followed by specific language such as “proceed with RALP” or “benefit from surgical”, thereby increasing its precision.


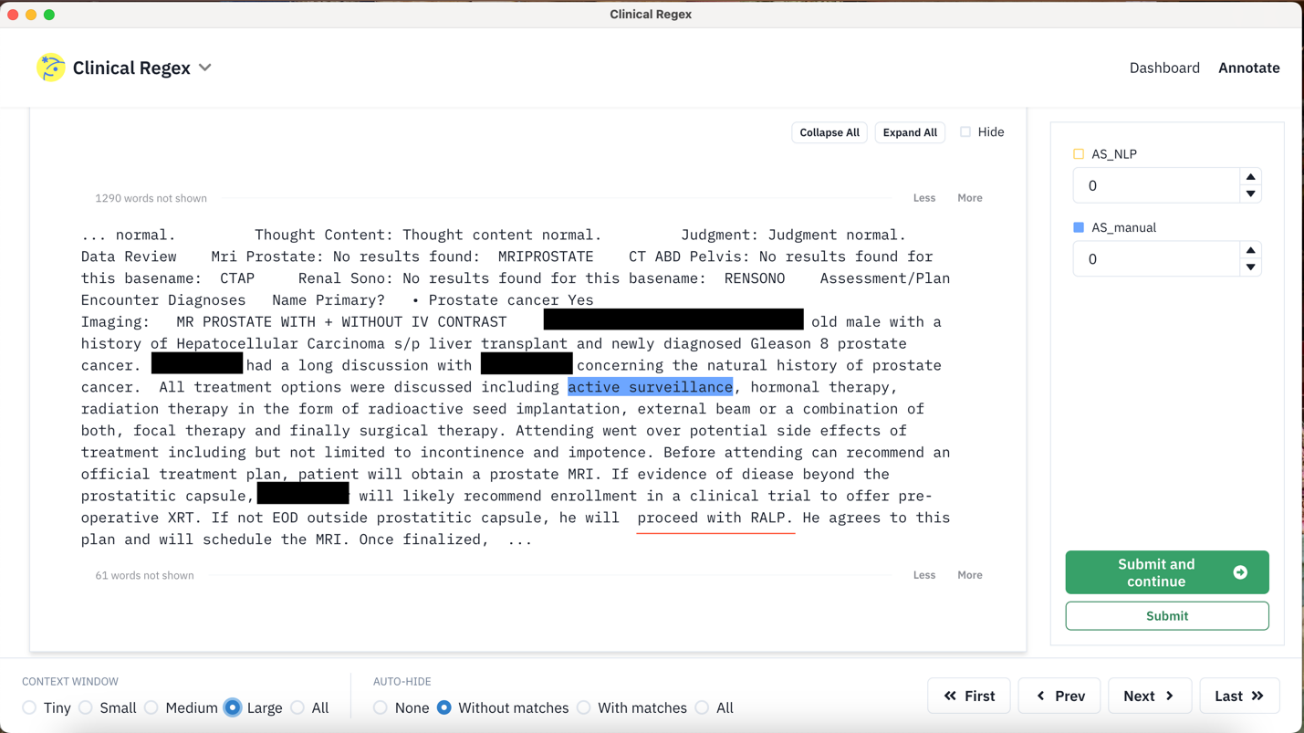


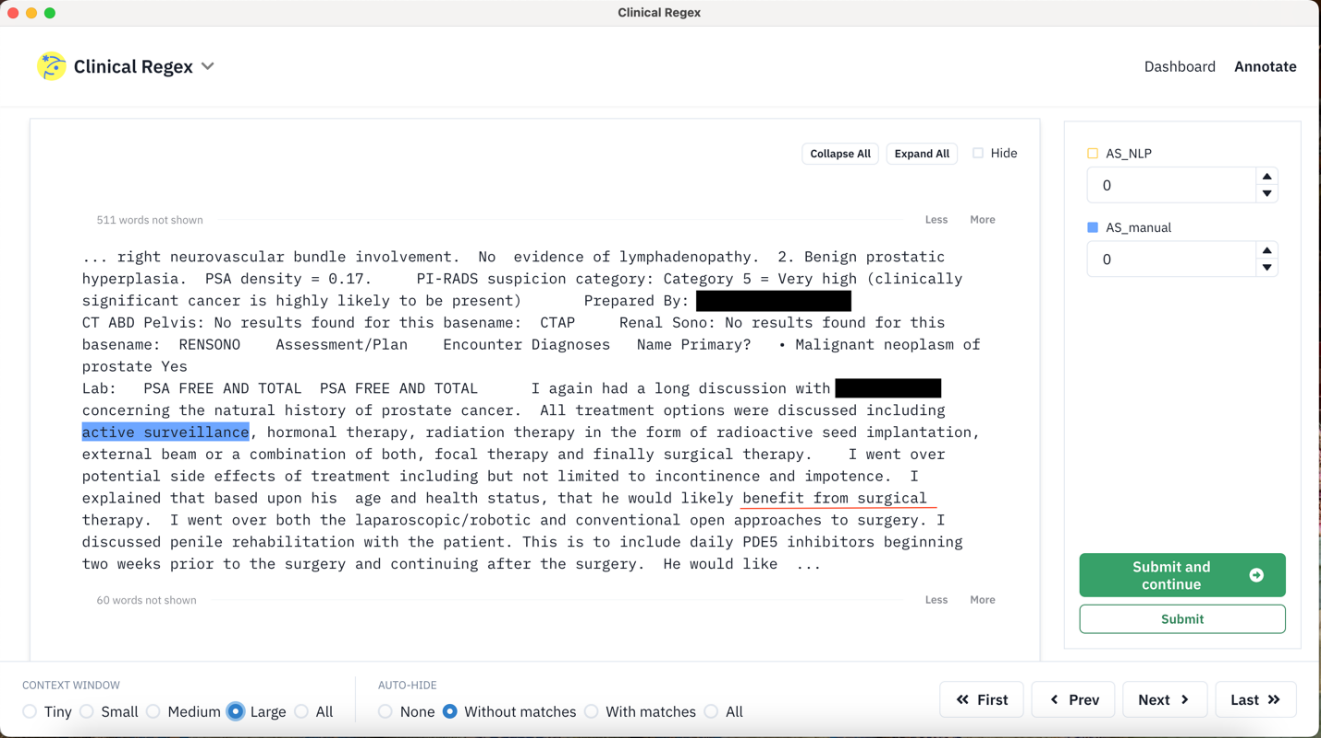


1. Human annotator uncertainty

When any of the annotators encountered an example in which there was uncertainty, these notes would be flagged and reviewed by the research team. Below is an example in the erectile function domain, where although erectile function is mentioned in the note, it does not specify whether it was discussed.


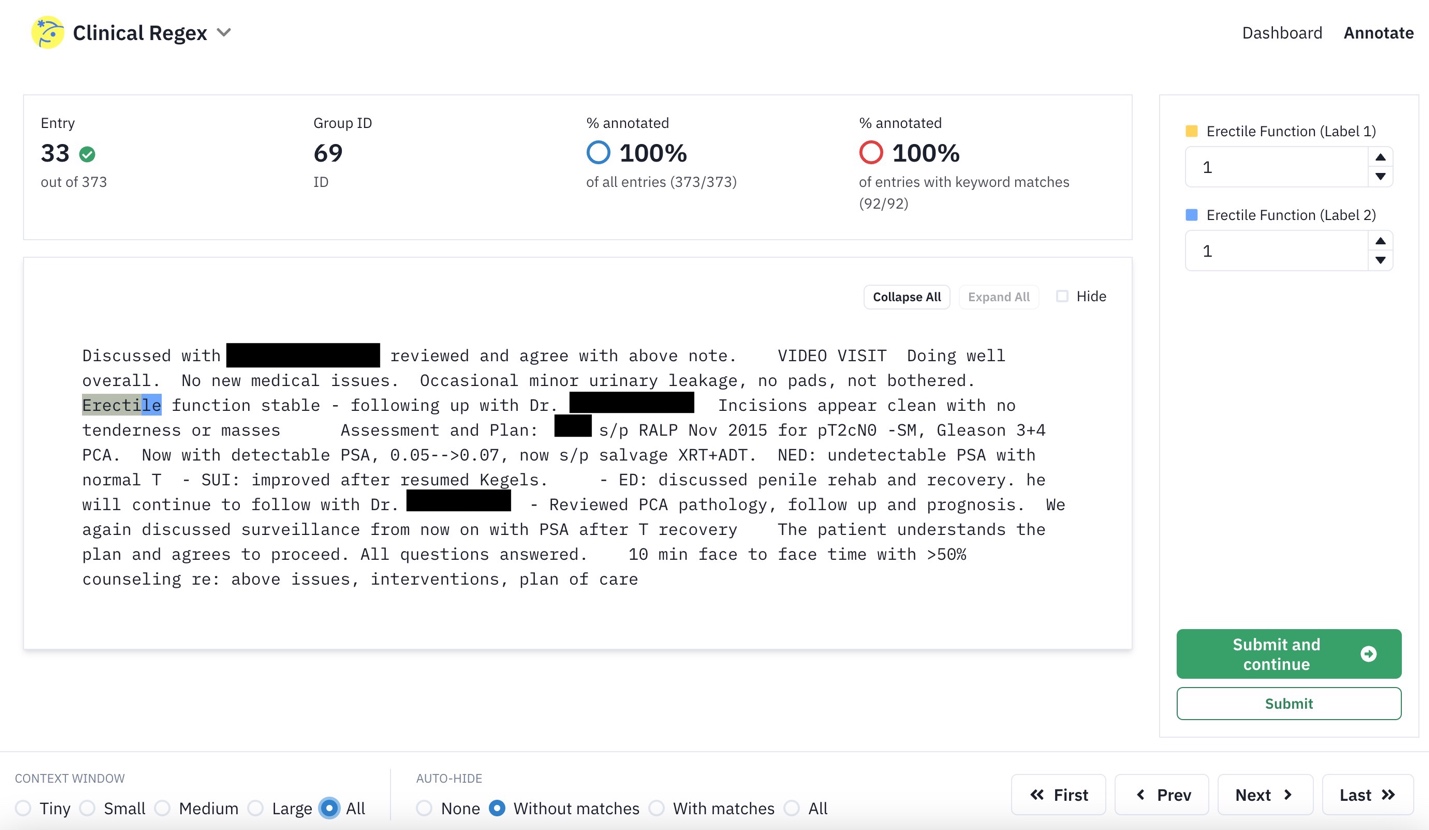


For this specific example, this was deemed to pertinent for the discussion of erectile function as it was being addressed with another provider and thus considered addressed for the patient in general.

**REFERENCES**

1. Schaeffer EM, Srinivas S, Adra N, et al. NCCN Guidelines® Insights: Prostate Cancer, Version 1.2023. *J Natl Compr Canc Netw*. 2022;20(12):1288-1298. doi:10.6004/jnccn.2022.0063

2. Genomic Testing for Urological Cancers. Decipher. Published 2023. Accessed November 28, 2023. https://decipherbio.com/

3. GPS Physician. mdxhealth. Accessed November 28, 2023. https://mdxhealth.com/gps-physician/

4. Prolaris® Prostate Cancer Prognostic Test. Myriad Genetics. Accessed November 28, 2023. https://myriad.com/genetic-tests/prolaris-prostate-tumor-test/

5. Barry MJ, Fowler FJ, O’Leary MP, et al. The American Urological Association symptom index for benign prostatic hyperplasia. The Measurement Committee of the American Urological Association. *J Urol*. 1992;148(5):1549-1557; discussion 1564. doi:10.1016/s0022-5347(17)36966-5

6. Rosen RC, Cappelleri JC, Smith MD, Lipsky J, Peña BM. Development and evaluation of an abridged, 5-item version of the International Index of Erectile Function (IIEF-5) as a diagnostic tool for erectile dysfunction. *Int J Impot Res*. 1999;11(6):319-326. doi:10.1038/sj.ijir.3900472

7. Chang P, Szymanski KM, Dunn RL, et al. Expanded Prostate Cancer Index Composite for Clinical Practice (EPIC-CP): Development and Validation of a Practical Health-Related Quality of Life Instrument for Use in the Routine Clinical Care of Prostate Cancer Patients. *J Urol*. 2011;186(3):10.1016/j.juro.2011.04.085. doi:10.1016/j.juro.2011.04.085

8. Lindvall C, Deng CY, Moseley E, et al. Natural Language Processing to Identify Advance Care Planning Documentation in a Multisite Pragmatic Clinical Trial. *J Pain Symptom Manage*. 2022;63(1):e29-e36. doi:10.1016/j.jpainsymman.2021.06.025
