# Supplementary material for: Ascertaining provider-level implicit bias in electronic health records with rules-based natural language processing: A pilot study in the case of prostate cancer
Source: PLoS One. 2024 Dec 30;19(12):e0314989. doi: 10.1371/journal.pone.0314989 (PMC11684669; doi:10.1371/journal.pone.0314989)
Supplement: Attachment — Submitted filename: PLOS Reviewer Comments_Final.docx [file pone.0314989.s002.docx]

Reviewer #1: The authors have submitted a thoughtful and compelling study. It is novel, thoughtful, and merits publication.

The authors examined whether implicit biases among providers affect treatment decisions in prostate cancer care using natural language processing analysis of medical records. Using case records of three urologic oncologists to over 1,000 patients, they analyzed care quality and intensity in four domains: active surveillance, molecular biomarker discussion, and evaluations of urinary and sexual function. The providers took an Implicit Association Test to measure racial biases. The authors offer that the implicit association test results showed that two of the providers did not have implicit bias favoring White patients while one did. The 4 care criteria provided differed for two of the providers in statistically significant ways, one of which was the provider with implicit bias. These findings again emphasize that CaP care may reflect aspects of the provider rather than the patients and that implicit biases might be included as such a factor. While IAT testing has gained in its use, most have recognized that the results reflect beliefs rather than actions. This paper adds an important perspective by looking at such actions (care provided).

The authors appropriately argue that these results are appropriately a pilot study and highlights the need for further research- as well as potential interventions- to address biases in care.

I offer suggestions, both major and minor, that might be helpful to the manuscript.

Major:
1. Three providers: The N for the study is really 3 providers rather than 1000+ patients.
a. Recommend that this be expressed as the first limitation in discussion rather than the fourth.

Thank you for your comments. We agree and have modified our manuscript to reflect this observation as the first limitation in **lines 286-289**.

2. IAT raw scores: If we are speaking about the same IAT, the test generates scores on a continuous scale that are subsequently categorized (-3 to 3)
a. The raw scores may be informative for Providers A and B, even if they are in the 0-0.15 range
b. The authors should state specifically that these numeric scores were either negative (a Black implicit bias) or positive numbers (a White implicit bias)

Thank you for your comment. We administered the publicly available IAT test available through Project Implicit. This website summarizes – but does not generate – D-scores (from -2 to 2) which adjust for variations between a participant’s reaction time by calculating the difference in reaction time between separate blocks of the IAT. These D-scores are typically extrapolated into a 7-point implicit bias scale (e.g., -0.15 to +0.15 indicating no preference; +0.16 to +0.34 as slight preference), which may be recategorized, as you described, to -3 to +3.^1^ Given we did not perform any regression analysis using IAT results in this pilot study of three providers, we found it sufficient to report summary IAT statistics (e.g., ‘slight’, ‘moderate’) and not especially informative to calculate raw D-scores through a complicated data collection process involving third-party software.^2^

3. Non-White versus Black: The relationship between the independent variable IAT testing (White/Black bias) as a predictor of the dependent variable- patient-by-patient care provided as analyzed by NLP analysis are not matched (White/Non-White).
a. In addition to those self-identifying as Black, exactly who is non-White? Can this be further broken down?

Thank you for this comment. We were able to further break down the tables to specifically identify those identifying as Black. We were unable to further separate Non-White into other subgroups due to a decision to limit collation of granular racial designations (see response to 3b, e.g., Egyptian, Bahrain, etc.) and our conservative methodology in racial designation to avoid misattribution.^3^

i. Can the analysis also be run for just patients who have identified as Black and White?

We re-ran the analysis for those patients identified as White vs. Black (shown below). Notably, our conclusions remain similar to the results presented in our paper. We believe our choice in comparing “White” vs. “non-White” better answers our study question as it is based on a research recommendation to understand race as a proxy for exposure to racism,^4^ which in the United States has historically been driven by White supremacy whereby “non-White” patients all share minority group status.

b. The breakdown of populations into “white” and “non-white” has a troublesome history (one-drop theory, social hierarchy, justification for slavery, etc.).

Thank you for the candid nature of this comment. Indeed, the use of the racial category “non-White” has a troublesome history as the reviewer alludes to here. However, we would argue that all racial categories are artificial, social constructs that may poorly reflect the experiences of the categorized peoples. For example, the category “Asian” includes a vast heterogeneous aggregation of Chinese, Indian, Filipino, Vietnamese, Korean, Japanese, Pakistani, Cambodian, Hmong, Thai, Laotian, Bangladeshi, Burmese, Nepalese, Indonesian, Sri Lankan, Malaysian, Bhutanese, Mongolian, and other racial/ethnic identities.^5^ Similar issues arise with use of the categories “Hispanic”,^6^ “Black”,^7^ and “White”.^8^ Our choice in comparing “White” versus “non-White” was based on a recommendation to understand race not as a measure of biologic difference, but rather as a proxy for exposure to racism,^4^ which in the United States has historically been driven by White supremacy.^9^

i. I do not believe that this is what the authors are implying, and a brief description of what is NOT meant could be in the limitations.

We have included this discussion in the limitations to address this observation in **lines 304-312**.

Minor:
1. Self-identified race of the providers: We learn some facts about the 3 providers, but not about their race
a. We learn years of experience and a metric of publication impact (h-factor), which perhaps may have been included to suggest that experienced scientists cannot be biased. This potentially can be included in the limitations section.

Thank you for your comment. We chose not to include provider self-identified race in the manuscript to ensure that the providers cannot be easily identified. Indeed, for this reason, we have elected to remove *h*-index and years in practice.

b. Interestingly for a publication about race and bias, we do not learn the self-identified race of the individuals. This is not meant to assign an explanation for their bias, but it allows a very brief mention in the discussion as to who mainly provides care to Black patients (and to point out that having more providers- and a diverse sample-in future studies allows for a deeper inquiry of racial concordant/racial discordant care).

Thank you for this comment. For reasons discussed in the previous comment, we have elected to not report provider self-identified race in the manuscript. However, we have discussed the importance of providing information regarding race-concordant care in future – and larger – studies of implicit bias in the Discussion in **line 283**.

2. SES variables: We have income, but not education. Educational attainment of the patients could be important (bias against low education, educational attainment being a confounder)
a. Education might be an important variable. If not available for analysis, that should be indicated as a limitation.

Thank you for your comments. We agree and have modified our manuscript to reflect the absence of educational attainment in our last limitation in line 291.

3. More about GG: GG1 and GG2 could be more explicitly defined as this article may be read widely and outside of urological circles

Thank you for this comment. We have included this description about the grade group designation for prostate cancer in **lines 77-80**.

4. IAT results: More information needed.
a. Knowing that the IAT results were not known to the providers would be important

Thank you for this observation. We noted in **lines 132-133** that the IAT results were not known to the providers prior to data collection was performed.

b. Likely the IAT test was done once with each provider. At what point was this done? That bias at one testing session is a predictor for care over 12+ years is a difficult issue and simply can be discussed as a limitation.

The IAT test was administered after data collection for this study was performed. We agree that assessment of implicit bias at one point in time may not be a predictor of care for the period reflected by our data and is included in the limitations under **lines 301-304**.

i. It may have been done late in the timeframe of the cohort, meaning that one’s attitude in 2020 was used to predict care delivered in 2010

As discussed in the previous comment, we have included this as a limitation under **lines 301-304**.

ii. Bias may not be constant over time (we hope it gets better!)

As discussed in the previous comment, we have included this as a limitation under **lines 301-304**.

5. NLP will likely not be able to verify to what extent shared decision making actually took place. We're relying on what the provider included in the note to represent this. This is a limitation.

Thank you for this comment. We have added that NLP will not be able to verify to what extent shared decision making took place in **lines 296-300** about the inability for NLP to capture characteristics of the patient encounter that are not explicitly documented. However, as previously noted, this is a limitation that exists even with manual chart review.

6. Getting a PSA test is also subject to bias: Everyone in this study already has a “positive” PSA value. That every patient getting into this analysis passed that barrier might suggest that the effect of bias in CaP care is likely larger than just these 4 criteria used (i.e., the need to consider the entire cascade of care framework).

Thank you for this insightful comment. We agree that every patient included in this analysis passed the barrier of being screened for prostate cancer screening and that it would be ideal to consider the effects of bias across the spectrum of prostate cancer care. Additional metrics we considered included: inquiry about family history, recommending MRI prior to pursuing biopsy, time from biopsy to treatment, etc. Given our data source, we chose the four quality indicators used in the manuscript as a small subset of what could possibly be evaluated in this and other conditions; we hope our study will enable such study in the future.

Reviewer #2: Ramaswamy et al. present data on implicit bias evaluation in prostate cancer patients using natural language processing. I´ve really enjoyed reading this manuscript and the authors should be congratulated for this really good use NLP. However, sample size (3 providers, 1000 patients) and granularity of data is limited.

Major issue #1:
One of the significant findings was that " Provider C recommended active surveillance (p<0.01, ϕ=0.175) and considered biomarkers (p=0.047, ϕ=0.127) more often in White men than expected, suggestive of treatment imparity". I would consider caution on that conclusion. Afro-Americans are often diagnosed at younger age and have more aggressive disease. I would recommend to re-run the analysis applying a more granular approach on race/ethnicity (Caucasian, Hispanics, Afro-American, Others).

Thank you for this comment. We specifically used the language of “more often than expected” in our conclusions as it is the statistical meaning of a chi-square test of independence. Regarding racial disaggregation, we provided further analysis and comments to address a similar concern in our responses to Reviewer 1 above.

Major issue #2:
Because MRI fusion biopsy cause artificial stage migration to some degree, I was wondering, if the proportion of fusion biopsy was equal in the different population or if this was considered during analysis. There are some works, finding lower rates of MRI fusion biopsy in disadvantaged population. Because active surveillance is more safe in an MRI fusion biopsy pathway, AS is more frequently recommended if a fusion biopsy was performed. This could have introduced a systematic bias.

Thank you for this interesting comment. To address the issues raised in this comment, it is important to individually address the presuppositions raised by the reviewer. First, it is contested whether MRI fusion biopsy leads to stage migration, i.e., increased rate of diagnosis of clinically significant prostate cancer (GG2+) in cases otherwise considered insignificant (GG1). One study nearly 1,300 patients who received MRI targeted biopsies demonstrating GG2 showed only a 2.6% rate of downgrading on final radical prostatectomy pathology.^10^ Additionally, in a recent population-based trial of 6,500 men, use of MRI targeted biopsies decreased the rate of overdiagnosis of clinically insignificant prostate cancer without increasing the detection of clinically significant prostate cancer when compared to systematic biopsy.^11^ While we agree that the literature demonstrates lower rates of MRI fusion biopsy in non-White populations,^12–14^ there is conflicting evidence that suggests active surveillance is more safe when MRI targeted biopsy is utilized^15–17^ or that providers would recommend active surveillance more frequently if a MRI targeted biopsy was performed.^18,19^ While utilization of MRI and MRI targeted biopsy are interesting quality indicators in their own rite, we do not did not evaluate it separately as a variable in our analysis for the reasons mentioned above, in no small part because it never was raised as an issue in any of the provider notes that were analyzed through NLP-screened human annotation.

Major issue #3:
Did you adjust the analysis for the year of counselling? (biomarker and AS were more implemented in recent years) Did you adjust the analysis on socioeconomic status and insurance coverage of biomarkers etc?

Thank you for this comment. We did not adjust the analysis for years of counselling because we did not perform a regression analysis based on time and rather provided an analysis across the entire study period. We have recognized this in our limitations in **lines 296-300**.

We did not adjust for socioeconomic status or insurance coverage for biomarkers given that the criteria for fulfilling any of the quality indicators were related to the “discussion” of the quality indicator in question with the patient (e.g., discussion of biomarkers) rather than eventual healthcare utilization which may be impeded by issues outside of the provider-patient relationship.

Minor issue #1:
I think it should be mentioned in the limitations that all providers were highly proliferated trained academics in a large cancer center which might influenced results. In rural areas, these differences might be more pronounced.

Thank you for this comment. We have included in this sentiment in the first limitation under **lines 286-289** that the providers included in this study work at an urban, tertiary-care, academic hospital which may influence our findings generalizability to other populations.

Minor issue #2:
I would recommend to introduce sub-section in the methods parts to make it more reader-friendly.

Thank you for this comment. We have introduced sub-sections to the methods section to ensure easier readability.

Minor issue #3:
I would recommend to add all tables / figures at the end to make it more easy to access them.

Thank you for this comment. We have adhered to PLOS One guidelines regarding presenting tables/figures.

**REFERENCES**

1.	Haider AH, Schneider EB, Sriram N, et al. Unconscious race and class bias: its association with decision making by trauma and acute care surgeons. *J Trauma Acute Care Surg*. 2014;77(3):409-416. doi:10.1097/TA.0000000000000392

2.	Tp C, R P, C P, et al. Survey-software implicit association tests: A methodological and empirical analysis. *Behavior research methods*. 2019;51(5). doi:10.3758/s13428-019-01293-3

3.	Freed GL, Bogan B, Nicholson A, Niedbala D, Woolford S. Error Rates in Race and Ethnicity Designation Across Large Pediatric Health Systems. *JAMA Network Open*. 2024;7(9):e2431073. doi:10.1001/jamanetworkopen.2024.31073

4.	Lett E, Asabor E, Beltrán S, Cannon AM, Arah OA. Conceptualizing, Contextualizing, and Operationalizing Race in Quantitative Health Sciences Research. *Ann Fam Med*. 2022;20(2):157-163. doi:10.1370/afm.2792

5.	Shah NS, Kandula NR. Addressing Asian American Misrepresentation and Underrepresentation in Research. *Ethn Dis*. 30(3):513-516. doi:10.18865/ed.30.3.513

6.	Abraído-Lanza AF, Mendoza-Grey S, Flórez KR. A Commentary on the Latin American Paradox. *JAMA Network Open*. 2020;3(2):e1921165. doi:10.1001/jamanetworkopen.2019.21165

7.	Pinheiro PS, Medina H, Callahan KE, et al. Cancer mortality among US blacks: Variability between African Americans, Afro-Caribbeans, and Africans. *Cancer Epidemiology*. 2020;66:101709. doi:10.1016/j.canep.2020.101709

8.	Read JG, Lynch SM, West JS. Disaggregating Heterogeneity among Non-Hispanic Whites: Evidence and Implications for U.S. Racial/Ethnic Health Disparities. *Popul Res Policy Rev*. 2021;40(1):9-31. doi:10.1007/s11113-020-09632-5

9.	Bailey ZD, Krieger N, Agénor M, Graves J, Linos N, Bassett MT. Structural racism and health inequities in the USA: evidence and interventions. *Lancet*. 2017;389(10077):1453-1463. doi:10.1016/S0140-6736(17)30569-X

10.	Martini A, Touzani A, Mazzone E, et al. Overdiagnosis and stage migration of ISUP 2 disease due to mpMRI-targeted biopsy: facts or fictions. *Prostate Cancer Prostatic Dis*. 2022;25(4):794-796. doi:10.1038/s41391-022-00606-6

11.	Hugosson J, Godtman RA, Wallstrom J, et al. Results after Four Years of Screening for Prostate Cancer with PSA and MRI. *New England Journal of Medicine*. 2024;391(12):1083-1095. doi:10.1056/NEJMoa2406050

12.	Hoge C, Verma S, Lama DJ, et al. Racial disparity in the utilization of multiparametric MRI–ultrasound fusion biopsy for the detection of prostate cancer. *Prostate Cancer Prostatic Dis*. 2020;23(4):567-572. doi:10.1038/s41391-020-0223-5

13.	Abashidze N, Stecher C, Rosenkrantz AB, Duszak R Jr, Hughes DR. Racial and Ethnic Disparities in the Use of Prostate Magnetic Resonance Imaging Following an Elevated Prostate-Specific Antigen Test. *JAMA Network Open*. 2021;4(11):e2132388. doi:10.1001/jamanetworkopen.2021.32388

14.	Leapman MS, Dinan M, Pasha S, et al. Mediators of Racial Disparity in the Use of Prostate Magnetic Resonance Imaging Among Patients With Prostate Cancer. *JAMA Oncology*. 2022;8(5):687-696. doi:10.1001/jamaoncol.2021.8116

15.	Ma TM, Tosoian JJ, Schaeffer EM, et al. The Role of Multiparametric Magnetic Resonance Imaging/Ultrasound Fusion Biopsy in Active Surveillance. *European Urology*. 2017;71(2):174-180. doi:10.1016/j.eururo.2016.05.021

16.	Tran GN, Leapman MS, Nguyen HG, et al. Magnetic Resonance Imaging–Ultrasound Fusion Biopsy During Prostate Cancer Active Surveillance. *European Urology*. 2017;72(2):275-281. doi:10.1016/j.eururo.2016.08.023

17.	Hamoen EHJ, Hoeks CMA, Somford DM, et al. Value of Serial Multiparametric Magnetic Resonance Imaging and Magnetic Resonance Imaging–guided Biopsies in Men with Low-risk Prostate Cancer on Active Surveillance After 1 Yr Follow-up. *European Urology Focus*. 2019;5(3):407-415. doi:10.1016/j.euf.2017.12.008

18.	Liu J, Womble PR, Merdan S, et al. Factors Influencing Selection of Active Surveillance for Localized Prostate Cancer. *Urology*. 2015;86(5):901-905. doi:10.1016/j.urology.2015.08.024

19.	Cher ML, Dhir A, Auffenberg GB, et al. Appropriateness Criteria for Active Surveillance of Prostate Cancer. *Journal of Urology*. 2017;197(1):67-74. doi:10.1016/j.juro.2016.07.005
